# SHARPIN Negatively Associates with TRAF2-Mediated NFκB Activation

**DOI:** 10.1371/journal.pone.0021696

**Published:** 2011-07-29

**Authors:** Yanhua Liang

**Affiliations:** Department of Dermatology, Yale University School of Medicine, New Haven, Connecticut, United States of America; Wellcome Trust Centre for Stem Cell Research, United Kingdom

## Abstract

NFκB is an inducible transcriptional factor controlled by two principal signaling cascades and plays pivotal roles in diverse physiological processes including inflammation, apoptosis, oncogenesis, immunity, and development. Activation of NFκB signaling was detected in skin of SHAPRIN-deficient mice and can be diminished by an NFκB inhibitor. However, *in vitro* studies demonstrated that SHARPIN activates NFκB signaling by forming a linear ubiquitin chain assembly complex with RNF31 (HOIP) and RBCK1 (HOIL1). The inconsistency between *in vivo* and *in vitro* findings about SHARPIN's function on NFκB activation could be partially due to SHARPIN's potential interactions with downstream molecules of NFκB pathway. In this study, 17 anti-flag immunoprecipitated proteins, including TRAF2, were identified by mass spectrum analysis among *Sharpin-Flag* transfected mouse fibroblasts, B lymphocytes, and BALB/c LN stroma 12 cells suggesting their interaction with SHARPIN. Interaction between SHARPIN and TRAF2 confirmed previous yeast two hybridization reports that SHARPIN was one TRAF2's partners. Furthermore, luciferase-based NFκB reporter assays demonstrated that SHARPIN negatively associates with NFκB activation, which can be partly compensated by over-expression of TRAF2. These data suggested that other than activating NFκB signaling by forming ubiquitin ligase complex with RNF31 and RBCK1, SHARPIN may also negatively associate with NFκB activation via interactions with other NFκB members, such as TRAF2.

## Introduction

While the protein, SHAPRIN (SHANK-associated RH domain interacting protein), has been known about for a decade, its role in causing immune disease and inflammation is just now becoming appreciated. SHARPIN in the inflammatory process triggers formation of the linear ubiquitin ligase complex (LUBAC). Lack of the *Sharpin* gene and therefore the protein (SHARPIN) leads to TNF-dependent inflammation of organs, particularly the skin, characterized as chronic proliferative dermatitis with keratinocyte death [1]–[3]. Mice deficient in *Sharpin* develop a severe autoinflammatory disorder with NFκB activation, in the absence of infectious agents, autoantibodies, or antigen-specific autoreactive T-cells [4]. This multiorgan-involved disorder was first described in a spontaneous, autosomal recessive mutation in mice and was named the chronic proliferative dermatitis mutation (CPDM, current allele symbol: *Sharpin^cpdm^* [5]–[6]. Affected mice exhibit clinical and molecular similarities to the idiopathic hypereosinophil syndrome in humans with clonal expansion of B1-B cells and CD3^+^CD4^−^CD8^−^ T cells [7]. Affected mice develop severe, scaly, red, ulcerated skin lesions with alopecia with systemic problems including immune system dysfunction, disorganization of secondary lymphoid organs, hepatosplenomegaly, infertility due to vaginal atresia, multiorgan granulocytosis, severe granulopoiesis, and other abnormalities [8]–[10]. SHARPIN is an important protein involved with multiple molecular pathways.

The human SHARPIN protein was first identified as a novel postsynaptic density protein and recently was shown to play a role in carcinogenesis [11]–[12]. However, no specific disease has yet been found associated with mutations or polymorphisms in the human *SHARPIN* gene. Full-length SHARPIN is predicted to be a protein of 380 amino acids with the exon 8 splice variant encoding 305 amino acids. Protein structural analysis suggested it had a highly conserved ubiquitin superfamily domain, implying that it is important in protein ubiquitination [13]–[14]. Protein sequence BLAST revealed significant homology with E3 ubiquitin ligase at the C terminal of SHARPIN. Protein ubiquitination and phosphorylation are two fundamental regulatory post-translational modifications controlling intracellular signaling events [15]–[16]. Ubiquitination of proteins involves the concerted action of the E1 ubiquitin-activated enzyme, E2 ubiquitin-conjugated enzymes, and E3 ubiquitin-protein ligases. Furthermore, the C-terminal region of SHARPIN shares significant amino acid sequence identity with the N-terminal region of RanBP-type and C3HC4-type zinc finger containing 1 (RBCK1, also known as HOIP), a protein known to function as a protein kinase C (PKC) binding protein as well as a transcriptional activator [11]. SHARPIN has significant sequence homology of its C-terminal region, enclosing a ubiquitin-like (UBL) domain and a ubiquitin-binding NPL4 zinc-finger domain (NZF), with the N-terminal region of RANBP (synonym:HOIL1L). SHARPIN was identified as a third component of the linear ubiquitin chain assembly complex (LUBAC), recruited to the CD40 and TNF receptor signaling complexes together with its other constituents, RBCK1 (HOIL1) and RNF31 (HOIP) [1]–[3]. Upon binding to the LUBAC subunit RNF31, SHARPIN stimulates the formation of linear ubiquitin chains *in vitro* and *in vivo*. Coexpression of SHARPIN and RNF31 promotes linear ubiquitination of IKBKG(synonym: NEMO) and subsequent activation of NFκB signaling. TRAF2 is a common signal transducer for TNFR and CD40 that mediates activation of NFκB [17]. A yeast two-hybrid (Y2H) screen with TRAF2 (TNF receptor-associated factor 2) as the bait (UCSD Nature Signaling Gateway, data center, yeast two-hybrid, http://www.signaling-gateway.org/data/Y2H/cgi-bin/y2h_int.cgi?id=53738) identified SHARPIN (alternative symbol: protein kinase C-interacting protein RBCC like 1 (RBCKL1)) as one of 8 preys in B cell lines. Primary structural analysis indicates that SHARPIN may interact with TRAF2 by protein ubiquitination as an E3 ligase.

Interleukin 1 (IL1)-mediated activation of NFκB signaling was shown to be one of the pathways responsible for chronic proliferative dermatitis in *Sharpin^cpdm^* mutant mice and an inhibitor of NFκB signaling, bortezomib, can significantly alleviate the skin problems [7]. NFκB signaling regulates diverse and key cellular and organismal processes including proliferation, differentiation, cell survival, apoptosis, immunity, and inflammation [18]–[20]. Dysregulation of the NFκB pathway, either by mutation or epigenetic mechanisms, is involved in many human and animal diseases, especially ones associated with chronic inflammation, immunodeficiency, or cancer [21]. The transcription factor NFκB is a complex formed by homodimerization and heterodimerization of the NFκB family members p50 (NFκB1), p52 (NFκB2), RelA (p65), RelB, and c-Rel [22]. NFκB is usually located in the cytoplasm bound to inhibitor of NFκB (IKB) proteins. However, diverse ligands, including IL1, tumor necrosis factor-α (TNFA) or lipopolysaccharide (LPS), bind to their receptors, and subsequently recruit cytoplasmic components including TRAFs, such as TRAF2 and TRAF6, to form a transmembrane complex. Ubiquitination of TRAFs will recruit and activate the IKK kinase complex, which in turn induces phosphorylation, ubiquitination, and subsequent degradation of the IKBs. The free NFκB can then translocate into the nucleus to control various transcriptional programs.

We show here that SHARPIN interacts with TRAF2, negatively associates with NFκB activation, and this can be partially compensated by over expression of TRAF2 *in vitro*.

## Methods

### Plasmid construct

Regions encompassing nucleotide 372 to 1538 of the full-length *Sharpin* cDNA (Thermo Scientific, Pittsburgh, PA) were PCR amplified with the forward primer 5′-GCC**AAGCTT**CTATCACGGAAGCACTCTCG-3′ and the reverse primer 5′-GAC**GAATTC**CTA*CTTATCGTCGTCATCCTTGTAATC*GGTGGAAGCTGCAGCAAGA-3′ (nucleotides in bold indicating the restriction sites for *Hind*III and *EcoR*I respectively). *Flag* sequence, in italics, was inserted before the stop codon of the *Sharpin* coding sequences. The resulted PCR product was sequenced to confirm no introduced mutations, digested with *Hind*III and *EcoR*I (New England Biolabs, Ipswich, MA) in a 50 µl reaction containing 1 µg of DNA incubated for 16 hours at 37 C and then subcloned into *Hind*III/*EcoR*I –cut pcDNA™3.1(+) (Invitrogen, Carlsbad, CA) creating pcDNA™3.1(+)/*Sharpin*-*Flag* construct. The junction sites including 227 nucleotides of *Sharpin*, 24 nucleotides of Flag, and 223 nucleotides of pcDNA™3.1(+) were sequenced to confirm the right orientation of the construct. The inserted fragments included the entire coding sequence from full-length cDNAs of *Sharpin*.

### Protein purification, immunoprecipitation, and mass spectrum analysis

After three passages, the MEF cultures were homogenous and the cells were transferred into 12-well culture dishes (5×10^5^) and grown in DMEM medium supplemented with 10% FBS for 24 h until they reached confluency. The cells were then transfected with 1.6 µg of constructs described above by Lipofectamine™ 2000 (Invitrogen, Carlsbad, CA). After 48 h, the culture medium was replaced by DMEM containing 500 µg/mL G418 (Invitrogen) for 2 weeks in order to eliminate non-transgenic cells [23]. Once confluent, cells were harvested to evaluate RNA expression, protein purification, and reporter assays.

Purification of SHARPIN-FLAG fusion protein was conducted using lysis buffer provided with the FLAG® tagged protein immunoprecipitation kit (Sigma, St. Louis, MO). Briefly, 1×10^6^ cells were resuspended in 1 ml lysis buffer supplemented with 10 µL protease inhibitor cocktail (Sigma), incubated on a shaker for 30 minutes, and centrifuged at 12,000 g for 10 minutes. Supernatants were stored at −70 C or on ice for immediate use.

FLAG tagged SHARPIN and its binding partners were immunoprecipitated using an ANTI-FLAG M2 affinity gel. Briefly, 40 µL of gel were placed into an empty chromatography spin column and the resin was collected by centrifugation then washed. The whole cell lysate was incubated with resin in the column on a shaker at 4 C overnight. Unbound proteins were washed away and the binding proteins were eluted in 20 uL of the sample buffer. FLAG-BAP protein provided by the kit was used as the positive control and MEFs transfected with the empty pcDNA™3.1 vector were used as the experimental negative control. Immunoprecipitated proteins were fractionated using a Criterion XT Bis-Tris gel (Bio-Rad, Hercules, CA). The gel was stained with Coomassie Brilliant Blue R-250 Dye (Thermo Scientific, Rockford, IL) and bands were cut off for mass spectroscopy.

### Immunoblotting

Whole cell extracts (WCE) or immunoprecipitated proteins were fractionated using Criterion XT Bis-Tris gel and electrophoretically transferred onto immune-blot polyvinylidene difluoride (PVDF) membranes (Bio-Rad, Hercules, CA). The blots were then incubated for 1 h with primary antibodies (anti-FLAG (Sigma)) at 2.5 µg/ml in blocking buffer (5% nonfat dry milk, 0.05% Tween-20 in TBS). Membranes were then incubated with horseradish peroxidase conjugated goat anti-rabbit IgG (Thermo Scientific) at 1∶2000 in 0.05% Tween-20 in TBS for 1 h. The blots were developed using enhanced ECL substrate detection kit (PerkinElmer, Waltham, MA) and then exposed using LAS-1000 plus (Fujifilm AG, Dielsdorf, Switzerland). Membranes were reprobed with anti β-Tubulin (Cell Signaling, Danvers, MA) at 1∶1000 as the experiment control.

### shRNA-mediated interference

Mouse embryonic fibroblasts (MEFs) (3×10^5^) were placed into each well of a 12-well plate the day before viral infection. When cells reached 50% confluency, 1.5×10^6^ mouse SHARPIN shRNA lentiviral particles (Santa Cruz, Santa Cruz, CA) containing 3 target-specific constructs were used to infect cells in Fb medium with 10 µg/ml polybrene (Santa Cruz) [24]. The culture medium was replaced with Fb medium without polybrene 12 h postinfection. Parallel experiments were performed using cop green fluorescence protein (GFP) lentiviral particles to monitor and optimize transduction efficiency. Cells infected with lentiviral particles were selected in 2 µg/mL puromycin dihydrochloride 48 h postinfection for a period of 3 days [25].

### NFκB luciferase reporter assays

For NFκB luciferase assays, MEFs were seeded at 70% confluency in 6-well plates and transfected with Lipofectamine 2000 reagent according to manufacturer's instructions (Invitrogen). Cells were transiently transfected with 0.25 µg/well with the NFκB-dependent luciferase reporter plasmid 4×κBL (a gift from Dr. Bill Sugden, University of Wisconsin-Madison, WI). Empty pcDNA™3.1(+) plasmid was used to balance the plasmid DNA amount in parallel experiments. For the luciferase assay, cells were lysed in reporter lysis buffer (Promega, Madison, WI) and activity was measured with the luciferase assay reagent (Promega) according to manufacturer's instructions. Normalization for transfection efficiency was done by co-transfecting 500 ng of a beta-galactosidase expression plasmid (pGK-beta-gal) and measuring beta-galactosidase activity. Relative luciferase activities are expressed as fold of activation over the activity of NFκB-dependent luciferase reporter alone and were calculated by dividing the values of luciferase activity with the values for beta-galactosidase activity. Three independent experiments were performed for each group.

### Quantitative PCR

Relative semi-quantitative PCR (QPCR) was performed per QuantiTect® SYBR® Green PCR kit from Qiagen by using an Applied Biosystems 7500 DNA sequence detection system (PerkinElmer Corp., Santa Clara, CA). The glyceraldehyde-3-phosphate dehydrogenase (*Gapdh*) gene was used as the control for the calculation of delta CT. Primer sequences were as follows: *Sharpin*: (f) 5′-CTCTTCATCGTCTGCCCATGT-3′, (r) 5′-TGATCCTGAAGGGCTGCAA-3′ (108 bp); *Gapdh*: (f) 5′-CCTCGTCCCGTAGACAAAATG-3′, (r) 5′-TCTCCACTTTGCCACTGCAA-3′ (100 bp). All reactions were performed in triplicate. RT-PCR data were analyzed by using the 2^−(ddCT)^ method as described previously [26].

### Statistical Analysis

Data were analyzed by using one-way analysis of variance (ANOVA). Differences between groups were determined with the Dunnett's multiple comparisons test and the data were expressed as (mean±standard error of the mean). Significance of differences was taken as the level of p<0.05.

## Results

### SHARPIN negatively relates to NFκB activation

**Figure 1:**
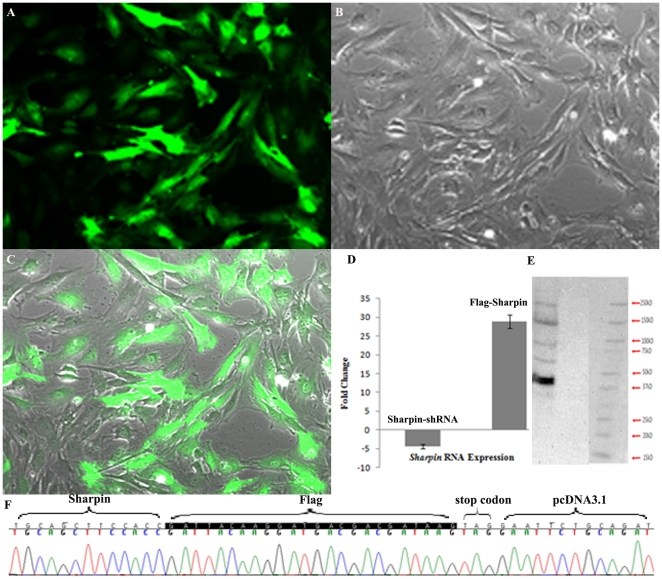
*Sharpin* expression in mouse embryonic fibroblasts. Transfection of *Flag* tagged *Sharpin* construct or shRNA lentivirus infection were respectively applied to mouse fibroblasts. Green fluorescent protein (GFP) labeled lentivirus were used as a control to monitor the infect efficacy. (**A**) Fluorescence detection of MEFs with co-transfection of both *Sharpin* shRNA lentivirus and GFP labeled lentivirus; (**B**) Differential interference micrograph; (**C**) Merged image of A and B; (**D**) Real-time PCR quantification of *Sharpin* in MEFs infected by *Sharpin*-shRNA or transfected by *Sharpin*-flag construct; (**E**) Anti-FLAG Western blot after anti-FLAG affinity gel immunoprecipitation of extracts from *Sharpin*-flag transfected MEFs. (**F**) Sequence data for the *Flag* tagged *Sharpin* construct.

**Figure 2:**
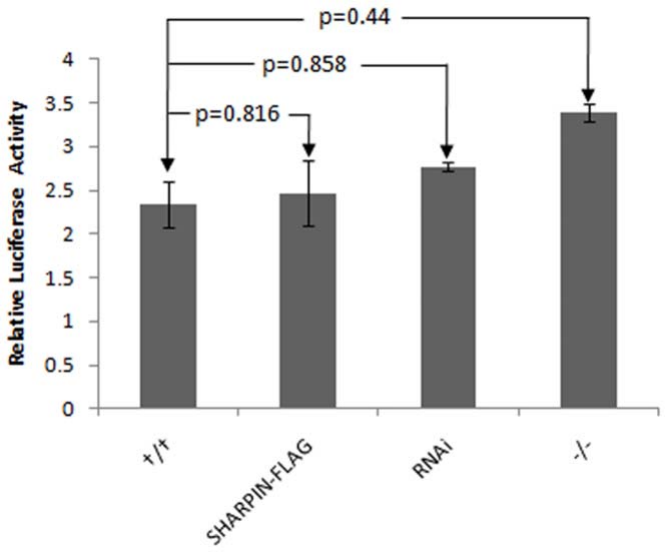
SHARPIN is negatively related to NFκB activation. Decreased *Sharpin* expression by shRNA interference or Loss of function of *Sharpin^cpdm^* mutation resulted in NFκB activation. On the contrary, overexpression of *Sharpin* by flag tagged *Sharpin* has inhibition effect on NFκB activation.

As reported previously, the transcription of most of the molecules in NFκB signaling is upregulated in the skin of *Sharpin^cpdm^* mutant mice with loss of function of SHARPIN. The severity of the skin lesions can be reduced with bortezomib, an NFκB inhibitor, implicating that the activation of NFκB signaling plays a key role in the skin phenotype caused by the loss of SHARPIN function [7]. To test whether endogenous SHARPIN functions as an inhibitory factor of NFκB, complete defective SHARPIN (*Sharpin^cpdm^* mutation), SHARPIN RNA interfered with shRNA lentivirus (**Fig. 1A–1C**), and over-expression of SHARPIN in a *Sharpin-Flag* construct were used to determine the effects of SHARPIN on NFκB activity. *Flag* tag was added before the stop codon of SHARPIN to avoid affecting SHARPIN's function (**Fig. 1F**). An NFκB (p65)-dependent luciferase reporter plasmid was transfected into MEFs already transfected with either a mouse specific shRNA lentiviral particles or Flag tagged *Sharpin* expression plasmids and *Sharpin^cpdm^* mutant MEFs. Expression of *Sharpin* mRNA was decreased by 4 fold by RNAi knockdown and increased by 28 folds in *Sharpin-Flag* construct transfected cells (**Fig. 1D**). As shown in **Fig. 2**, although no significant differences were observed, the DNA binding and transcriptional activities of NFκB (p65) were marginally augmented by reduction of *Sharpin* expression, reinforcing the notion that *Sharpin* acts as a negative regulator of the NFκB pathway. Over-expression of *Sharpin* mRNA did not significantly affect the NFκB transcriptional activity, which might be controlled by a feedback circuit.

### SHARPIN regulates NFκB activation through TRAF2 involvement

To determine the direct or indirect role of SHARPIN on NFκB activation, the *Sharpin-Flag* construct was transfected into MEF, B cells, and BLS12 cells. Whole cell extracts were immunoprecipated using a anti-FLAG affinity gel. After immunoprecipitation, anti-FLAG antibody was used in Western blots to detect the existence and amount of SHARPIN-FLAG fusion protein (**Fig. 1E**). Western blots revealed 5 hybridized bands among which the 45 kDa band, indicative of the SHARPIN-FLAG fusion protein, had the strongest intensity. Immunoprecipitated proteins using the anti-FLAG affinity gel, were separated by 2D-PAGE gel. Bands were analyzed by mass spectroscopy. Seventeen proteins were identified from B cells, MEF, and BLS12 cells (**Table 1**). Five proteins were identical in all cells:FYN binding protein (FYB), sodium channel, voltage-gated, type XI, alpha (SCN11A), zinc finger protein 106 (ZFP106), serine/threonine kinase 24 (STK24), and cut-like homeobox 1 (CUX1). Four proteins, TRAF2, TEKT4, NSD1, and ASAP2, were identified as SHARPIN partners in B cells and BLS12 cells but not in MEFs. TRAF2 was previously shown to bind to SHARPIN in yeast-two hybridization studies (UCSD Nature Signaling Gateway). Data presented here confirmed this interaction between SHARPIN and TRAF2. CCAAT/enhancer binding protein, alpha (CEBPA) and proteasome 26S subunit, non-ATPase, 1 (PSMD1) interacted with SHARPIN in both BLS12 and MEF cells while adenylate cyclase activating polypeptide 1 (ADCYAP1), ring finger protein 20 (RNF20),apoptosis-inducing factor, mitochondrion-associated 1 (AIFM1) were unique partners in B cells. Uubiquitin carboxy-terminal hydrolase L1(UCHL1), surfeit gene 6 (SURF6), and calpain 13 (CAPN13) were identified only in BLS12 cells as SHARPIN partners.

**Table 1:** Proteins uniquely associated with anti-FLAG affinity gel purified SHARPIN as determined by mass spectrometry analysis in various cells.

Symbol	MGI ID	B cell	BLS12	MEF	Function
FYB	1346327	**Yes**	**Yes**	**Yes**	TCR-mediated NFκB activation
SCN11A	1345149	**Yes**	**Yes**	**Yes**	neurotrophin-evoked depolarization
ZFP106	1270153	**Yes**	**Yes**	**Yes**	transcription regulation
STK24	2385007	**Yes**	**Yes**	**Yes**	protein (MAPK1/MAPK3) phosphorylation
CUX1	88568	**Yes**	**Yes**	**Yes**	cell migration and invasion
TRAF2	101835	**Yes**	**Yes**	No	NFκB activation
TEKT4	1919090	**Yes**	**Yes**	No	sperm motility
NSD1	1276545	**Yes**	**Yes**	No	apoptosis
ASAP2	2685438	**Yes**	**Yes**	No	regulation of ARF GTPase activity
CEBPA	99480	No	**Yes**	**Yes**	arrest cell proliferation
PSMD1	1917497	No	**Yes**	**Yes**	proteasome complex
ADCYAP1	105094	**Yes**	No	No	neural stem cell proliferation, platelet activation
RNF20	1925927	**Yes**	No	No	E3 ligase activity
AIFM1	1349419	**Yes**	No	No	apoptosis, apoptotic mitochondrial changes
UCHL1	103149	No	**Yes**	No	Regulation of synaptic structure
SURF6	98447	No	**Yes**	No	DNA binding and rRNA processing
CAPN13	2685789	No	**Yes**	No	apoptosis, cell division, synaptic plasticity

**Figure 3:**
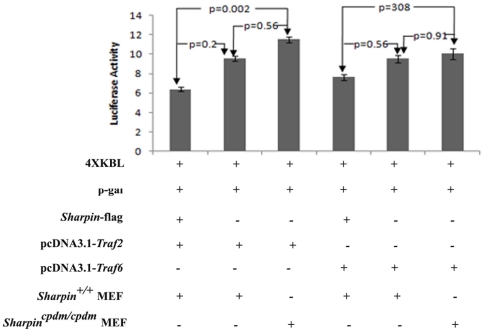
Role of SHARPIN on TRAF2-mediated NFκB activation. *Sharpin*-flag, pcDNA-*Traf2*, pcDNA-*Traf6* were respectively transfected into wildtype or *Sharpin^cpdm/cpdm^* mutant mouse embryonic fibroblasts using different combinations. After selection and confirmation, a luciferase labeled NFκB plasmid was transfected into the cells to quantify chang.

To further clarify the synergistic or antagonistic effect, *Sharpin*-*Flag* construct was co-transfected with or without pcDNA-*Traf*2/6 into wildtype or *Sharpin^cpdm^* mutant MEFs. As shown in **Fig. 3**, SHARPIN expression inhibited TRAF2-mediated NFκB activation markedly and had a weaker but no significant inhibitory effect (about 20% inhibition) on NFκB activation by TRAF6 compared with NFκB activation by pcDNA-*Traf2*/6 alone.

## Discussion

It has been two decades since the spontaneous mutant, chronic proliferative dermatitis, was reported. Although pathological changes have been well described [8], [27], this mouse model was not brought to the forefront until the mutated gene responsible was identified [6]. Little was known about the mutated gene, *Sharpin*, in any species [10]–[11], [28]–[29]. The systemic eosinophilic phenotypes in *Sharpin^cpdm^* mice was proposed as a potential model for human idiopathic hypereosinophilic syndrome based on laboratory and clinical comparisons although this has yet to be confirmed [7]. Activation of NFκB signaling in the skin was identified as the primary cause of the chronic proliferative dermatitis phenotype in *Sharpin^cpdm^* mutant mice and inhibition of NFκB activation by bortezormib can alleviate the skin phenotype suggesting a treatment for some forms of hypereosinophilic syndrome in humans.

The present study identified 17 putative proteins that interact with SHARPIN among which TRAF2 was previously identified in yeast-two hybrid studies. Reporter assays indicated that SHARPIN negatively associates with TRAF2-mediated NFκB activation. Although no significant decrease in NFκB activity was detected when cells were transfected with SHARPIN alone, co-transfection of SHARPIN and TRAF2 inhibited transcription of a luciferase-based NFκB reporter. It could be interpreted that NFκB activation was augmented *in vivo* in many types of cells in the SHAPRIN-deficient skin, although only marginal change was observed *in vitro* in mouse fibroblasts alone. By contrast, Ikeda *et al* demonstrated that coexpression of SHARPIN and RNF31 promoted linear ubiquitinization of IKBKG with subsequent activation of NFκB signaling [1]. It is possible that the activation of NFκB signaling in the SHARPIN and RNF31 coexpression system is partially due to RNF31 over-expression. It may potentially be interpreted as that SHARPIN could play opposite roles for NFκB signaling at different points in the gene network, activating NFκB signaling by forming linear ubiquitin chain assembly complex with RNF31, or inhibiting NFκB signaling through interaction with downstream NFκB members such as TRAF2. Alternatively, SHARPIN may function differently in different cell types which is supported by the differential results obtained in these studies *in vitro* as well as in preliminary studies using a *Shaprin* conditional null allele *in vivo* (Sundberg, unpublished data).

The *Shank* family of proteins are known as anchoring/scaffold proteins containing multiple protein–protein interaction sites that include ankyrin repeats, SH3 domain, PDZ domain, long proline-rich region, and SAM domain [30]. SHARPIN was identified as a new binding partner of *Shank* through the ankyrin repeat domain of *Shank1* [11]. SHARPIN is relatively abundantly expressed in multiple organs including brain, heart, and testis. It was previously proposed that *Sharpin* may have an additional function(s) besides a role as a scaffolding partner of *Shank1* [11]. Using TRAF2 as bait, SHARPIN was identified in B cells by yeast-two hybridization. In the study reported here, mass spectrum analysis identified 17 partners of SHARPIN from 3 types of cells, including mouse B cells, BLS12 cells, and fibroblasts. Among 5 widely expressed proteins of interaction with SHARPIN, FYB may bind to the SH domain of SHARPIN regulating NFκB activation by control of IkappaB kinase alpha/beta (IKKA/B) phosphorylation and IKKG ubiquitination [31]–[32]. *Sharpin* may play a role in carcinogenesis through the interaction with FYB, which binds to FYN, a newly identified oncogene [32]–[33]. TRAF2 interacts with SHARPIN in B cells and BLS12 cells, supporting the previous yeast-two hybridization report. As shown in figure 2, decreased expression or loss of function of SHARPIN can induce increased NFκB activity. SHARPIN had a significant negative effect on TRAF2-mediated NFκB activation and slightly affected TRAF6-mediated NFκB activation (Fig. 3).

Loss of *Sharpin* in mice results in marked epidermal hyperplasia with marked keratinocyte apoptosis, the latter of which is regulated through the mitochondrial pathway in a caspase-dependent manner [34]. Three of 17 SHARPIN's partners play important roles on apoptosis, including AIFM1, CAPN13, and NSD1. As AIFM1 is a key player of mitochondrial regulation of cell death, AIFM1 may be the most likely target of SHARPIN on keratinocyte death in *Sharpin^cpdm^* mice [35].

In conclusion, this study has identified proteins interacting with SHARPIN in several signaling pathways. The *Sharpin* gene has a negative effect on NFκB activation, probably through protein-protein interaction with TRAF2 and/or FYB. Whether SHARPIN eventually activates or inhibits NFκB signaling depends on its conclusive interactions at different points on NFκB pathway, mutations in different functional domains, or cell type. SHARPIN may also interact with AIFM1 to regulate mitochondria-mediated apoptosis in keratinocyte. The complex molecular functions of SHARPIN have been largely overlooked but the development and refinement of mouse models is this to be investigated.
